# Inflammation Associated With Obesity, Aging, and Amyloid Burden in Adults With Down Syndrome

**DOI:** 10.1002/oby.70229

**Published:** 2026-06-05

**Authors:** Victoria L. Fleming‐Batayneh, Brian C. Helsel, Lauren T. Ptomey, Benjamin L. Handen, Mark Mapstone, Melissa Petersen, Sharon J. Krinsky‐McHale, Christy L. Hom, Davneet Minhas, Weiquan Luo, Charles Laymon, Joseph H. Lee, Annie Cohen, Beau M. Ances, Adam M. Brickman, Margaret Pulsifer, H. Diana Rosas, Florencia Lai, Shahid H. Zaman, Elizabeth Head, Dana Tudorascu, Julie Price, Fredrick Schmitt, Bradley T. Christian, Ozioma Okonkwo, Sigan L. Hartley, for the Alzheimer Biomarkers Consortium‐Down Syndrome (ABC‐DS) Investigators, Beau M. Ances, Howard F. Andrews, Karen Bell, Rasmus M. Birn, Adam M. Brickman, Peter Bulova, Jeff Burns, Amrita Cheema, Kewei Chen, Bradley T. Christian, Isabel Clare, Ann D. Cohen, Eric W. Doran, Tatiana M. Foroud, Benjamin L. Handen, Jordan Harp, Sigan L. Hartley, Elizabeth Head, Denise Head, Christy Hom, Lawrence Honig, Milos D. Ikonomovic, Sterling C. Johnson, M. Ilyas Kamboh, David Keator, Julia K. Kofler, William Charles Kreisl, Sharon J. Krinsky‐McHale, Florence Lai, Patrick Lao, Charles Laymon, Joseph Hyungwoo Lee, Ira T. Lott, Victoria Lupson, Mark Mapstone, Davneet Singh Minhas, Neelesh Nadkarni, Sid O'Bryant, Deborah Pang, Melissa Petersen, Julie C. Price, Lauren Ptomey, Margaret Pulsifer, Michael S. Rafii, Herminia Diana Rosas, Frederick Schmitt, Nicole Schupf, Wayne P. Silverman, Dana L. Tudorascu, Rameshwari Tumuluru, Badri Varadarajan, Michael A. Yassa, Shahid Zaman, Fan Zhang

**Affiliations:** ^1^ Waisman Center, University of Wisconsin‐Madison Madison Wisconsin USA; ^2^ Department of Neurology University of Kansas Medical Center Kansas City Kansas USA; ^3^ Department of Internal Medicine University of Kansas Medical Center Kansas City Kansas USA; ^4^ Department of Psychiatry University of Pittsburgh Pittsburgh Pennsylvania USA; ^5^ Department of Neurology University of California Irvine School of Medicine Irvine California USA; ^6^ Department of Family Medicine Institute for Translational Research University of North Texas Health Science Center Fort Worth Texas USA; ^7^ Department of Psychology New York Institute for Basic Research in Developmental Disabilities Staten Island New York USA; ^8^ Department of Psychiatry and Human Behavior University of California Irvine California USA; ^9^ Department of Radiology University of Pittsburgh Pittsburgh Pennsylvania USA; ^10^ Department of Bioengineering University of Pittsburgh Pittsburgh Pennsylvania USA; ^11^ Taub Institute for Research on Alzheimer's Disease and the Aging Brain, Sergievsky Center, and Department of Neurology, Vagelos College of Physicians and Surgeons, Columbia University New York New York USA; ^12^ Behavioral Health Service Line, VA Pittsburgh Healthcare System Pittsburgh Pennsylvania USA; ^13^ Department of Neurology Washington University School of Medicine St. Louis Missouri USA; ^14^ Department of Psychiatry Massachusetts General Hospital, Harvard Medical School Boston Massachusetts USA; ^15^ Department of Psychiatry University of Cambridge Cambridge UK; ^16^ Department of Pathology and Laboratory Medicine University of California Irvine School of Medicine Irvine California USA; ^17^ Department of Radiology Massachusetts General Hospital, Harvard Medical School Boston Massachusetts USA; ^18^ Department of Neurology University of Kentucky Lexington Kentucky USA; ^19^ Department of Psychiatry University of Wisconsin‐Madison Madison Wisconsin USA; ^20^ Department of Medical Physics School of Medicine and Public Health, University of Wisconsin‐Madison Madison Wisconsin USA; ^21^ Department of Geriatrics and Gerontology School of Medicine and Public Health, University of Wisconsin‐Madison Madison Wisconsin USA; ^22^ Department of Human Development and Family Studies University of Wisconsin‐Madison Madison Wisconsin USA

## Abstract

**Objective:**

Adults with Down syndrome (DS) often show elevated systemic inflammation, but the association with obesity, aging, and Alzheimer's disease (AD) pathology is not well understood.

**Methods:**

Data were drawn from 188 nondemented adults with DS participating in the Alzheimer Biomarkers Consortium‐DS (ABC‐DS). Participants completed clinical assessments, blood draws, and neuroimaging. Plasma biomarkers included indicators of general, pro‐, and anti‐inflammation. Mixed linear models tested associations between BMI, age, PET‐measured amyloid burden, and inflammatory biomarkers, adjusting for sex, trisomy type, and collection site. False discovery rate correction was applied.

**Results:**

The majority of the participants met criteria for obesity. Higher BMI was significantly associated with elevated levels of CRP, IL‐6, TNF‐α, B2M, IL‐18, and slCAM‐1 (*p* < 0.05). Older age was significantly associated with higher B2M (*β* = 1.22e + 05, *p* < 0.001). Amyloid burden was positively associated with IL‐6 (*β* = 0.005, *p* = 0.037).

**Conclusions:**

Obesity, aging, and amyloid burden relate to systemic inflammation in adults with DS. Obesity showed the strongest and most consistent associations, emphasizing the value of regular monitoring and weight management strategies to help reduce inflammation. Aging and early amyloid accumulation showed more limited links with systemic inflammation; future work should examine whether these processes are more closely related to biomarkers of neuroinflammation as AD progresses.

## 1 Introduction

Down syndrome (DS) is the most common genetic cause of intellectual disability (ID), affecting 1 in 700 to 1500 live births in the United States (US) [1, 2]. Individuals with DS show elevated levels of systemic inflammation (e.g., interleukin [IL]‐10, IL‐6, tumor necrosis factor‐alpha [TNF‐α]) compared to those without DS [3, 4]. Despite these findings, it remains unclear how metabolic factors, like body mass index (BMI) or the aging process, influence systemic inflammation in this population.

The majority of adults with DS have BMI in the overweight or obesity range, with estimates ranging from 60% to 90% [5, 6, 7]. In the general population, excess body fat is linked to chronic, low‐grade inflammation, often called metaflammation [8]. This commonly includes elevated levels of C‐reactive protein (CRP), IL‐6, IL‐18, and TNF‐α [9, 10, 11] but also includes soluble intercellular adhesion molecule‐1 (sICAM‐1) [12]. However, research in DS is limited and it remains unclear whether obesity is associated with the same profile of blood‐based biomarkers of inflammation [13, 14, 15]. Chromosome 21 contains genes that alter metabolic functioning [16, 17], suggesting that obesity is not related to inflammation in traditional ways. Indeed, prior studies indicate that obesity is not related to many cardiovascular conditions (e.g., high cholesterol) in individuals with DS, as is true outside of DS [18]. Nonetheless, in a mouse model of DS, Ts56Dn mice with higher adiposity had higher levels of IL‐6 than those with lower adiposity [13], suggesting that obesity may still be related to a more narrow profile of elevated inflammation even in the context of trisomy 21.

Adults with DS also experience signs of accelerated aging, including graying of the hair and skin wrinkling in their 30s [19, 20], cataracts in their 40s [21, 22], and earlier age of menopause [23, 24]. In the general adult population, aging is associated with elevated plasma biomarkers of inflammation including IL‐6, CRP, sICAM‐1, beta‐2 macroglobulin (B2M), and alpha‐2 macroglobulin (A2M) [25, 26]. This inflammaging is thought to be driven by the accumulation of cell damage, harmful byproducts leaking into surrounding tissues (i.e., oral and gut microbiota), and cellular senescence [27].

Additionally, nearly all adults with DS develop Alzheimer's disease (AD) pathology, with brain amyloid plaques observed in the 30s–50s [28, 29, 30] and dementia symptoms emerging in the early‐to‐mid 50s [31]. The high risk for AD in DS is linked to an overproduction of amyloid resulting from three copies of the amyloid precursor protein (APP) gene [32]. Studies on late‐onset AD in the general population show elevated inflammatory biomarkers associated with mild cognitive impairment (MCI) and AD dementia [33, 34, 35]. Elevated inflammation is thought to exacerbate the spread and deposition of amyloid plaques, contributing to neuronal damage leading to AD [36, 37]. In DS, systemic biomarkers of inflammation including eotaxin‐3, IL‐10, CRP, IL‐18, and serum amyloid A (SAA) have been found to distinguish between adults with DS with MCI or AD dementia from those who are cognitively stable [38, 39]. Whether blood‐based biomarkers of systemic inflammation correspond to early amyloid deposition during the preclinical AD stage (i.e., prior to MCI or dementia) has not been examined in DS but is particularly critical for informing DS‐associated AD screening and care.

The present study examines the relationship between obesity, aging, and preclinical stages of AD (i.e., early amyloid deposition) and systemic inflammation in adults with DS. Using RStudio, we modeled BMI, age, and PET‐assessed amyloid burden together while controlling for biological sex, trisomy type, and data collection site. We hypothesized that higher BMI would be associated with elevated inflammation (i.e., CRP, IL‐6, IL‐18, and TNF‐α), and adults with DS who have BMI in the overweight or obesity range would have higher inflammation than those with BMI in the normal range. Age was hypothesized to be positively related to inflammation, especially higher levels of IL‐6 and TNF‐α, which have been shown in the general population [40, 41]. Lastly, elevated inflammation (i.e., IL‐6) was expected to be related to higher early amyloid burden.

## 2 Methods

### 2.1 Participants

Data for this study were drawn from the Alzheimer Biomarkers Consortium‐DS (ABC‐DS; see Handen et al. [42]), a multisite natural history study of AD in DS. ABC‐DS includes 11 sites across the US, the UK, and Puerto Rico. Eligible participants were 25 years or older, had a karyotype‐confirmed diagnosis of DS (i.e., full trisomy, partial trisomy, or mosaic), had no contradictions for neuroimaging (e.g., pregnancy, metal implants), and had no untreated medical or psychiatric conditions that affect cognition. The ABC‐DS study protocol was approved by a central review board (Advarra Pro00044843), and written informed consent was obtained from all participants or their legal representatives. A single Internal Review Board (IRB) provided study oversight and the IRB at all local data collection sites approved the study.

The subsample of adults with DS included in the present study was those in the larger ABC‐DS (total *N* = 503; 201 [40.0%]) who had PET and BMI data. Of these 201 participants, 188 (93.5%) also had available plasma biomarkers of inflammation. This subsample (*n* = 188) was significantly younger than the overall cohort (*t*(501) = 6.392, *p* < 0.001) but did not differ in biological sex (*χ* ^2^ = 3.5711, *p* = 0.059) or race/ethnicity (*χ* ^2^ = 3.639, *p* = 0.603).

### 2.2 Procedures

Participants completed a multiday baseline visit that included a blood draw for karyotyping (or pulling medical records) and inflammatory biomarker assay, a comprehensive neuropsychological exam, and MRI and PET imaging for amyloid quantification (Centiloids). Study procedures were harmonized across all data collection sites through the use of detailed standard operational procedure manuals, certification processes, and site reliability checks [42].

### 2.3 Measures

#### 2.3.1 Sociodemographic Characteristics

Participant age was calculated using the participant's date of birth and the date of the visit, and the study partner reported on biological sex and race/ethnicity. Premorbid ID level was determined based on medical records or standardized IQ assessments (from the Stanford‐Binet Intelligence Scales, Fifth Edition [SB5] [43]) or the Kaufman Brief Intelligence Test, Second Edition (KBIT‐2) [44], prior to any concerns of MCI or dementia. Premorbid ID was classified as follows: mild (“mental age”: 9–14 years), moderate (“mental age”: 4–8 years), and severe/profound (“mental age”: < 4 years). Mental age equivalent scores were used to code premorbid ID level rather than standard IQ scores given the limited range in the SB5 abbreviated IQ score and KBIT‐2, which do not allow for the differentiation of moderate versus severe/profound ID.

Clinical AD status was determined through a clinical consensus review involving DS clinicians and trained staff, who were blind to neuroimaging and inflammation data. Data reviewed included medical history, clinical lab results, cognition, and behavioral and functional assessment [42]. Participants were classified as cognitive stable, indicating no concerns about cognitive or functional decline; MCI, indicating evidence of limited cognitive or functional decline; AD dementia, indicating evidence of substantial cognitive and functional decline; or unable to determine, meaning that due to a recent medical issue or significant stressful event (e.g., death of a caregiver, loss of employment), it was not possible to make a clinical status determination. Current analyses only included participants with DS who were deemed cognitively stable.

#### 2.3.2 BMI

Height and weight were measured with participants' shoes removed using a stadiometer or tape measure and digital scale. BMI was calculated as weight in kilograms divided by height in meters squared (kg/m^2^). BMI was classified into three categories: normal/underweight (< 25 kg/m^2^), overweight (25 to < 30 kg/m^2^), and obesity (≥ 30 kg/m^2^) [45].

#### 2.3.3 Plasma Inflammation Biomarker Assays

Samples were processed at the Institute for Translational Research (ITR) Biomarker Core. Proteomic assays were automated using the customized Hamilton Robotics StarPlus system, enhancing reliability by minimizing human error and reducing coefficients of variation (CV), which strengthened quality assurance and quality control (QA/QC). Any realiquoting was performed using the Hamilton easyBlood robotic system.

Commercially available assays were sourced from Meso Scale Discovery (MSD; http://www.mesoscale.com) and conducted using electrochemiluminescence (ECL) technology in accordance with previously published protocols [46, 47]. A total of 500 μL of plasma was used to measure the following biomarkers of general inflammatory (CRP, A2M, B2M), proinflammatory (TNF‐α, IL‐6, IL‐18, sICAM‐1), and anti‐inflammatory (IL‐10) proteins. All inflammation biomarkers were measured in picograms per milliliter (pg/mL).

#### 2.3.4 MRI and Amyloid PET

High‐resolution T1‐weighted MRI scans were acquired using either a 3D fast spoiled gradient echo (FSPGR) or a magnetization‐prepared rapid acquisition gradient echo (MPRAGE) sequence to provide anatomical reference for PET image processing. Specific details on the neuroimaging equipment and acquisition parameters are described in an ABC‐DS methods publication [42].

Amyloid burden was assessed using either [^11^C]‐Pittsburgh Compound B (PiB) or florbetapir (AV‐45). Imaging acquisition occurred 50–70 min post injection of radiotracer. Data were reconstructed using iterative methods and corrected for scanner dead time and radioactive decay. Images were collected in 5‐min frames and corrected for motion on a frame‐by‐frame basis prior to the calculation of an averaged single‐frame (50–70 min) image. The index of amyloid burden was quantified using the Centiloid method [48], which standardizes PET data across data collection sites. PET scans were registered to the corresponding T1‐weighted MRI, which was then normalized to the MNI152 template using SPM8 [49]. The transformation parameters were applied to the PET image to warp it to the MNI template. Radioactivity concentrations were extracted for both the standard Centiloid global cortical region and the whole cerebellum using regions of interest (ROI) predefined on the MNI152 template [48], available at The Global Alzheimer's Association Interactive Network website (https://gaain.org/centiloid‐project). Global standardized uptake value ratios (SUVr) were calculated as the ratio of tracer concentration in the global cortical ROI to the cerebellar reference region. These SUVr values were then converted to Centiloid units using linear‐plus‐constant transformations specific to each tracer (PiB [48] and florbetapir [49]).

### 2.4 Data Analysis

Prior to conducting primary analyses, data distributions were examined using histograms and descriptive statistics to assess normality and skewness (e.g., means and standard deviations [SD]). Extreme values exceeding ±4 SD from the mean were winsorized, setting them to the nearest ±4 SD limit to reduce their impact on the results. To verify robustness of the findings, additional analyses were performed after excluding these outliers.

To investigate the relationship between BMI, age, and early amyloid burden on inflammatory biomarkers in adults with DS, separate linear mixed models were fit for each inflammatory biomarker using the lme package in R [50]. For every linear mixed model the unstandardized beta, standard error (SE), 95% confidence interval (CI), *t* value, and *p* value were reported for all predictors and covariates. Each model included BMI, age, and amyloid burden as fixed effects, with biological sex and trisomy type as covariates and data collection site as a random effect. To correct for multiple comparisons, false discovery rate (FDR) was controlled using the Benjamini‐Hochberg method with a significant threshold of *q* < 0.05 [51]. In follow‐up analyses, models were re‐run testing biological sex as a moderator of the association between BMI and inflammatory biomarkers given evidence outside of DS of sex differences in the association between BMI and inflammation [52, 53].

## 3 Results

Participants ranged from 25 to 67 years of age (*M* = 40.4, SD = 8.8). The average BMI was 32.1 (SD = 7.8), with 105 (55.8%) individuals having obesity, 56 (29.8%) classified as overweight, and 27 (14.4%) with a normal BMI. About 49% of participants were female (*n* = 92). The majority identified as White (*n* = 178, 94.6%), while 2 participants identified as Black (1.1%), 3 identified as Asian (1.6%), and 5 had unknown racial identity (2.7%). Regarding premorbid ID level, 90 participants (47.9%) had mild ID, 84 (44.6%) had moderate ID, and 14 (7.5%) had severe ID. The majority had full trisomy 21 (168, 89.6%), while 7 (3.5%) had mosaic trisomy 21, 10 (5.4%) had translocation of chromosome 21, and 3 (1.5%) had a pattern that did not fit into these categories. These characteristics are summarized in Table 1.

**TABLE 1:** Participant characteristics.

Characteristic	*n* = 188^a^
Age, mean ± SD	40.4 ± 8.8
BMI, mean ± SD	32.1 ± 7.8
Biological sex	
Female	92 (49%)
Male	96 (51%)
Race/ethnicity	
White	178 (94.6%)
Black	2 (1.1%)
American Indian	0 (0%)
Asian	3 (1.6%)
Hispanic	0 (0%)
Unknown	5 (2.7%)
Premorbid ID	
Mild	90 (47.9%)
Moderate	84 (44.6%)
Severe	14 (7.5%)
Trisomy type	
Full trisomy	168 (89.6%)
Mosaicism	7 (3.5%)
Translocation	10 (5.4%)
Other	3 (1.5%)

^a^ Intellectual disability (ID) levels reflect the following “mental age”: mild, ≥ 9 years; moderate, 4–8 years; severe/profound, < 4 years.

Descriptive statistics showed that several plasma biomarkers were positively skewed to varying degrees, including CRP (*M* = 1.226e + 07 pg/mL, skewness = 2.43), IL‐6 (*M* = 1.172 pg/mL, skewness = 3.34), IL‐10 (*M* = 0.623 pg/mL, skewness = 5.38), TNF‐α (*M* = 3.055 pg/mL, skewness = 1.14), and B2M (*M* = 7.112e + 06 pg/mL, skewness = 1.17). However, a few biomarkers were approximately normally distributed, including A2M (*M* = 1.087e + 09 pg/mL, skewness = 0.97), IL‐18 (*M* = 105.711 pg/mL, skewness = 0.89), and sICAM‐1 (*M* = 3.613e + 05 pg/mL, skewness = 0.69). During follow‐up sensitivity analyses, extreme values, outliers exceeding ±4 SD, were identified and removed from each biomarker, including CRP (*n* = 3), IL‐6 (*n* = 1), IL‐10 (*n* = 3), TNF‐α (*n* = 2), and B2M (*n* = 1); no outliers were detected for A2M, IL‐18, and sICAM‐1.

### 3.1 BMI and Inflammation

**FIGURE 1:**
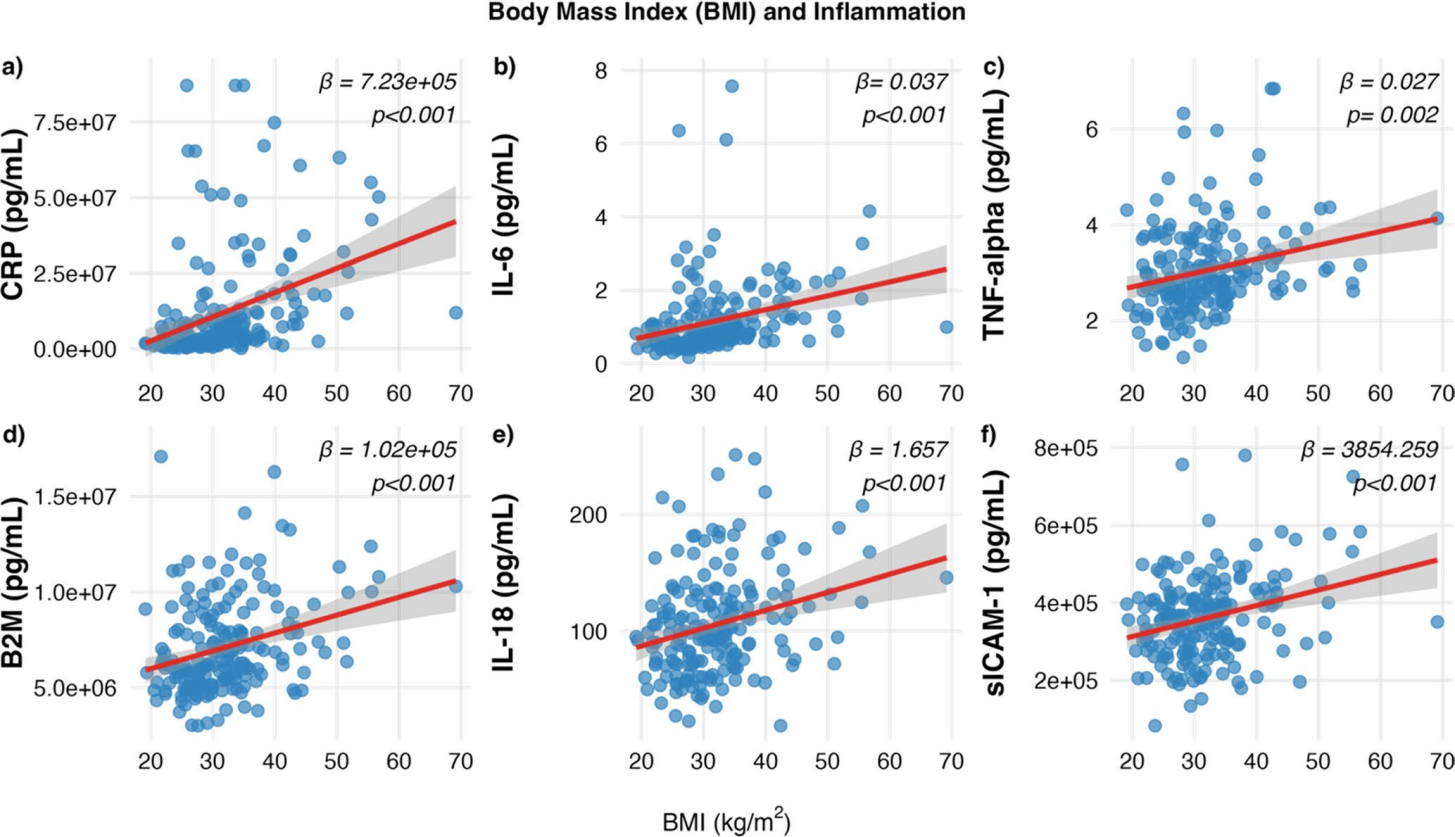
Associations between BMI and markers of inflammation. (a) C‐reactive protein (CRP; pg/mL), (b) interleukin‐6 (IL‐6; pg/mL), (c) tumor necrosis factor‐alpha (TNF‐alpha; pg/mL), (d) beta‐2 macroglobulin (B2M; pg/mL), (e) interleukin‐18 (IL‐18; pg/mL), (f) soluble intercellular adhesion molecule‐1 (sICAM‐1; pg/mL). Lines represent linear mixed‐effects regression models adjusted for age, amyloid burden, biological sex, trisomy type, and site (random effect). *β* coefficients and FDR‐corrected *p* values (Benjamini and Hochberg [51]) are displayed within panels. Extreme values exceeding ±4 SD were winsorized to the ±4 SD threshold prior to analysis; CRP (*n* = 3), IL‐6. (*n* = 1), TNF‐alpha (*n* = 2), and B2M (*n* = 1) were affected; no outliers were detected for IL‐18 or sICAM‐1. Sample size for this analysis was *n* = 188. [Color figure can be viewed at wileyonlinelibrary.com]

**FIGURE 2:**
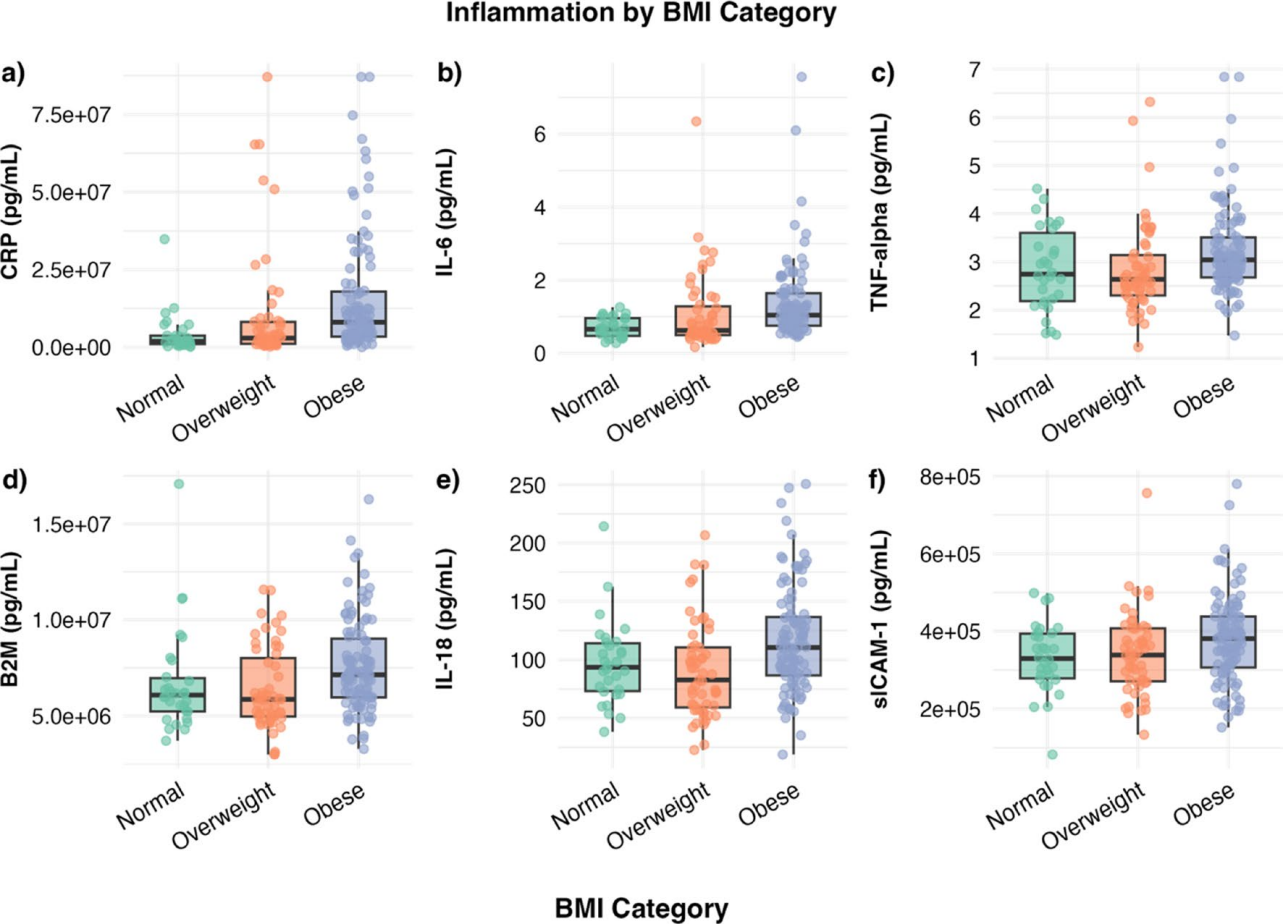
BMI and inflammation by category. (a) C‐reactive protein (CRP; pg/mL), (b) interleukin‐6 (IL‐6; pg/mL), (c) tumor necrosis factor‐alpha (TNF‐alpha; pg/mL), (d) beta‐2 macroglobulin (B2M; pg/mL), (e) interleukin‐18 (IL‐18; pg/mL), (f) soluble intercellular adhesion molecule‐1 (sICAM‐1; pg/mL). Box plots represent median and interquartile ranges (IQR); whiskers denote 1.5 IQR. Associations were evaluated using linear mixed‐effects models adjusting for covariates as described in Table 2. Extreme values exceeding ±4 SD were winsorized prior to statistical modeling; CRP (*n* = 3), IL‐6 (*n* = 1), TNF‐alpha (*n* = 2), and B2M (*n* = 1) were affected; no outliers were detected for IL‐18 or sICAM‐1. Sample size for this analysis was *n* = 188. [Color figure can be viewed at wileyonlinelibrary.com]

Linear mixed‐effects models assessed the association between BMI and each inflammatory biomarker, controlling for age, biological sex, trisomy type, and site (random). (Table 2). After FDR correction, higher BMI was positively associated with higher levels of CRP (*β* = 7.23e + 05, *p* < 0.001; Figure 1a), IL‐6 (*β* = 0.037, *p* < 0.001; Figure 1b), TNF‐α (*β* = 0.027, *p* = 0.002; Figure 1c), B2M (*β* = 1.02e + 05, *p* < 0.001; Figure 1d), IL‐18 (*β* = 1.657, *p* < 0.001; Figure 1e), ad sICAM‐1 (*β* = 3854.259, *p* < 0.001; Figure 1f). These positive associations are visually represented in the corresponding scatterplots (Figure 1a–f). In Figure 2a–f box plots illustrate these biomarkers across BMI categories. No significant associations were observed between BMI and IL‐10 or A2M.

**TABLE 2:** Multilevel models of BMI, age, and amyloid predicting inflammation.

Outcome	Fixed effects	*B*	SE	95% CI	*t*	*p*
CRP	Intercept	−3.20e + 07	9.44e + 06	[−5.05e + 07, −1.35e + 07]	−3.39	< 0.001
	BMI	7.23e + 05	1.61e + 05	[4.08e + 05, 1.04e + 06]	4.50	< 0.001
	Age	2.35e + 05	1.76e + 05	[−1.10e + 05, 5.80e + 05]	1.33	0.183
	Centiloid	−43624.370	52701.059	[−1.47e + 05, 59669.706]	−0.83	0.408
	Biological sex (female)	3.61e + 06	2.49e + 06	[−1.27e + 06, 8.49e + 06]	1.45	0.147
	Trisomy type (full trisomy)	6.01e + 06	2.26e + 06	[1.59e + 06, 1.04e + 07]	2.66	0.008
IL‐6	Intercept	−1.097	0.441	[−1.961, −0.233]	−2.49	0.014
	BMI	0.037	0.008	[0.022, 0.052]	4.94	< 0.001
	Age	0.011	0.008	[−0.005, 0.027]	1.33	0.186
	Centiloid	0.005	0.002	[0.000, 0.010]	2.10	0.037
	Biological sex (female)	0.156	0.117	[−0.073, 0.385]	1.34	0.183
	Trisomy type (full trisomy)	0.236	0.106	[0.028, 0.443]	2.23	0.027
IL‐10	Intercept	0.712	0.283	[0.157, 1.266]	2.51	0.013
	BMI	−0.007	0.005	[−0.017, 0.002]	−1.48	0.142
	Age	−0.000	0.005	[−0.011, 0.010]	−0.07	0.941
	Centiloid	−0.002	0.002	[−0.005, 0.002]	−0.96	0.339
	Biological sex (female)	0.079	0.075	[−0.068, 0.226]	1.05	0.294
	Trisomy type (full trisomy)	0.034	0.068	[−0.099, 0.167]	0.50	0.615
TNF‐α	Intercept	1.409	0.510	[0.409, 2.409]	2.76	0.006
	BMI	0.027	0.009	[0.010, 0.044]	3.12	0.002
	Age	0.014	0.010	[−0.004, 0.033]	1.52	0.131
	Centiloid	−0.003	0.003	[−0.008, 0.003]	−0.99	0.324
	Biological sex (female)	0.077	0.133	[−0.184, 0.339]	0.58	0.562
	Trisomy type (full trisomy)	0.100	0.120	[−0.136, 0.336]	0.83	0.406
A2M	Intercept	7.27e + 08	1.66e + 08	[4.01e + 08, 1.05e + 09]	4.37	< 0.001
	BMI	−3.14e + 06	2.71e + 06	[−8.46e + 06, 2.18e + 06]	−1.16	0.247
	Age	5.89e + 06	3.07e + 06	[−1.37e + 05, 1.19e + 07]	1.92	0.056
	Centiloid	−1.38e + 06	8.94e + 05	[−3.13e + 06, 3.71e + 05]	−1.55	0.122
	Biological sex (female)	1.46e + 08	4.18e + 07	[6.44e + 07, 2.28e + 08]	3.50	< 0.001
	Trisomy type (full trisomy)	4.81e + 07	3.75e + 07	[−2.54e + 07, 1.22e + 08]	1.28	0.200
B2M	Intercept	−1.96e + 06	1.21e + 06	[−4.33e + 06, 4.09e + 05]	−1.62	0.105
	BMI	1.02e + 05	20618.851	[61723.484, 1.43e + 05]	4.95	< 0.001
	Age	1.22e + 05	22517.806	[78249.705, 1.67e + 05]	5.44	< 0.001
	Centiloid	−6002.128	6757.592	[−19247.008, 7242.753]	−0.89	0.374
	Biological sex (female)	4.18e + 05	3.20e + 05	[−2.09e + 05, 1.05e + 06]	1.31	0.191
	Trisomy type (full trisomy)	2.91e + 05	2.90e + 05	[−2.78e + 05, 8.60e + 05]	1.00	0.316
IL‐18	Intercept	41.266	24.302	[−6.365, 88.897]	1.70	0.091
	BMI	1.657	0.414	[0.845, 2.469]	4.00	< 0.001
	Age	0.133	0.453	[−0.754, 1.020]	0.29	0.769
	Centiloid	0.155	0.136	[−0.111, 0.422]	1.14	0.254
	Biological sex (female)	0.324	6.434	[−12.287, 12.934]	0.05	0.960
	Trisomy type (full trisomy)	1.561	5.830	[−9.865, 12.988]	0.27	0.789
sICAM‐1	Intercept	1.44e + 05	59382.171	[28070.261, 2.61e + 05]	2.43	0.016
	BMI	3854.259	1008.770	[1877.070, 5831.448]	3.82	< 0.001
	Age	1896.277	1109.774	[−278.879, 4071.434]	1.71	0.090
	Centiloid	−473.030	331.338	[−1122.453, 176.393]	−1.43	0.155
	Biological sex (female)	19922.013	15640.049	[−10732.482, 50576.508]	1.27	0.204
	Trisomy type (full trisomy)	−4317.453	14149.633	[−32050.734, 23415.828]	−0.31	0.761

*Note: B* = unstandardized regression coefficient; SE = standard error. Large values are displayed in scientific notation for readability. All models corrected for multiple comparisons using the false discovery rate (Benjamini and Hochberg [51]). Sex reference = Male; site included as a random intercept.

Abbreviations: A2M, alpha‐2 macroglobulin; B2M, beta‐2 macroglobulin; CRP, C‐reactive protein; IL, interleukin; sICAM‐1, soluble intercellular adhesion molecule‐1; TNF‐α, tumor necrosis factor–alpha.

### 3.2 Age and Inflammation

**FIGURE 3:**
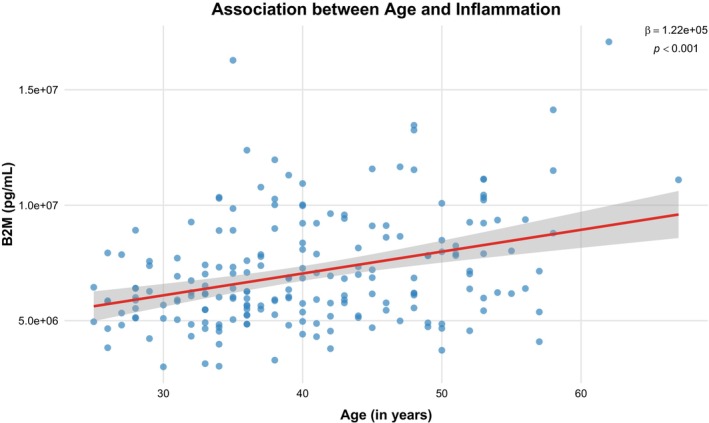
Associations between age and markers of inflammation. Graph represents the linear mixed‐effects regression model for age and beta‐2 macroglobulin (B2M; pg/mL) adjusted for BMI, amyloid burden, biological sex, trisomy type, and site (random effect). The *β* coefficient and FDR‐corrected *p* value (Benjamini and Hochberg [51]) are displayed. Extreme values exceeding ±4 SD were winsorized to the ±4 SD threshold prior to analysis; B2M had 1 affected value. Sample size for this analysis was *n* = 188. [Color figure can be viewed at wileyonlinelibrary.com]

Models examining age as a predictor of inflammation, controlled for BMI, biological sex, trisomy type, and site (random), revealed a significant positive association with B2M (*β* = 1.22e + 05, *p* < 0.001; Figure 3). A trend‐level positive association between age and A2M (*β* = 5.89e + 06; *p* = 0.056) was also revealed (Table 2). There were no significant associations between age and CRP, IL‐6, IL‐10, TNF‐α, IL‐18, and sICAM‐1.

### 3.3 Amyloid Burden and Inflammation

**FIGURE 4:**
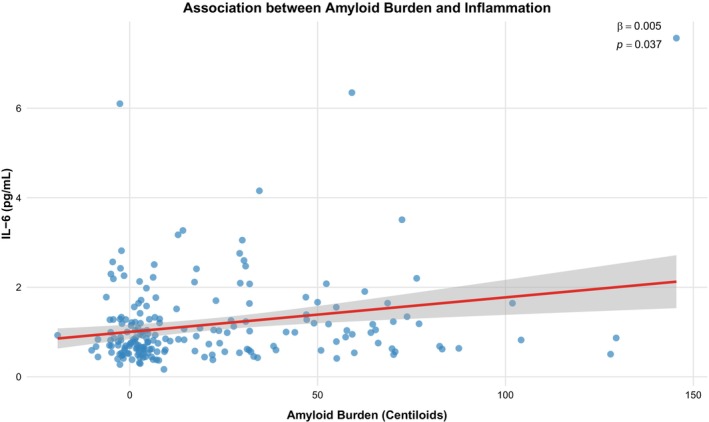
Associations between amyloid burden and markers of inflammation. Graph represents the linear mixed‐effects regression model for amyloid burden and interleukin‐6 (IL‐6; pg/mL) adjusted for BMI, age, biological sex, trisomy type, and site (random effect). The *β* coefficient and FDR‐corrected *p* value (Benjamini and Hochberg [51]) are displayed. Extreme values exceeding ±4 SD were winsorized to the ±4 SD threshold prior to analysis; IL‐6 had 1 affected value. Sample size for this analysis was *n* = 188. [Color figure can be viewed at wileyonlinelibrary.com]

Controlling for BMI, age, biological sex, trisomy type, and site (random), higher amyloid had a significant positive association with IL‐6 (*β* = 0.005, *p* = 0.037; Table 2 and Figure 4). There were no other significant associations.

### 3.4 Sensitivity Analysis and Follow‐Up Analyses

Follow‐up analyses excluding outliers confirmed the robustness of the primary findings (Table S1). After removing these outliers, BMI remained significantly positively associated with CRP (*β* = 7.96e + 05, *p* < 0.001), IL‐6 (*β* = 0.034, *p* < 0.001), TNF‐α (*β* = 0.025, *p* < 0.001), B2M (*β* = 1.06e + 05, *p* < 0.001), IL‐18 (*β* = 1.567, *p* < 0.001), and sICAM‐1 (*β* = 3331.667, *p* < 0.001). Age remained significantly positively associated with B2M (*β* = 1.09e + 05, *p* < 0.001) and additionally became significantly positively associated with IL‐6 (*β* = 0.015, *p* = 0.023). The prior association between amyloid burden and IL‐6 was no longer statistically significant, though the effect direction remained positive.

As shown in Table S1, the interaction of biological sex × BMI had limited effects on inflammatory biomarkers using the Winsorized dataset. There was a significant interaction of biological sex × BMI for B2M (*β* = 8.790e + 04, *p* = 0.0398) (Figure S1a) but not for other biomarkers. In a sensitivity analysis excluding ±3 SD (Table S2), the biological sex × BMI interaction was instead observed for CRP (*β* = 5.874e05, *p* = 0.0101) (Figure S1b).

## 4 Discussion

Individuals with DS are known to exhibit elevated systemic inflammation [3, 4], but the contribution of obesity, aging, and early AD pathology to this inflammation has been unclear. The triplication of genes that alter metabolic functioning associated with DS [16, 17] has shown a weakened association between obesity and certain cardiovascular conditions such as high cholesterol [18]. However, the current study demonstrates that higher BMI is associated with elevated systemic inflammation in adults with DS, in ways that mirror those in adults without DS. Specifically, elevated BMI was associated with higher levels of a wide array of inflammatory biomarkers, including CRP, IL‐6, TNF‐α, B2M, IL‐18, and sICAM‐1, consistent with patterns observed in the general adult population [54, 55]. The proinflammatory effects of increased adipose tissue, particularly visceral fat, are thought to arise from cytokine‐mediated macrophage activation, and they may contribute to insulin resistance outside of DS [8] and may act similarly to increase inflammation in DS. These findings underscore the clinical importance of monitoring BMI and metabolic health in adults with DS and implementing strategies to manage weight such as nutritional counseling and structured exercise programs that have been tailored for individuals with ID [56, 57].

In contrast, age had a more limited relationship with systemic inflammation in this sample. Older age was associated with elevated B2M and demonstrated a trend level toward increased A2M. Sensitivity analyses excluding outliers further revealed a positive relationship between age and IL‐6. Outside of DS, B2M has been linked to oxidative stress [58], frailty [59], and late‐onset AD [60]. In our sample, B2M was related to both older age and higher BMI. Thus, this inflammatory marker may reflect converging metaflammation and inflammaging pathways in adults with DS that involve immune activation and altered oxidative stress processes. Clinically, monitoring B2M alongside BMI and other metabolic biomarkers could help identify individuals at risk for age‐related inflammatory complications, potentially informing early preventive strategies. It should be noted that for a limited set of inflammatory biomarkers (B2M and CRP), higher BMI had a more pronounced association with elevated systemic inflammation for females than males with DS. Sex differences in adipose tissue distribution, particularly central visceral versus peripheral fat deposition, are associated with distinct inflammatory and metabolic outcomes outside of DS [52, 53]. Future research should further examine biological sex differences in inflammatory profiles in DS.

Previous studies in late‐onset AD have demonstrated that inflammation both contributes to and is triggered by amyloid deposition [36, 37]. However, given the 1.5× overexpression of the APP gene in DS that is associated with the increased risk for AD [32], it is not clear if early amyloid deposition is similarly tied to inflammation in DS. In the present study, early amyloid burden, measured prior to MCI or AD dementia, was positively associated with IL‐6, consistent with prior observations from late‐onset AD research [61]. The absence of associations between amyloid deposition and other inflammatory biomarkers may suggest that systemic inflammation may have limited utility for identifying preclinical AD in DS, prior to MCI and AD dementia. However, it is likely that early amyloid deposition has stronger ties to biomarkers specific to neuroinflammation, and those biomarkers could have greater clinical utility in AD screening. Thus, future work should examine the association between early amyloid burden and blood or cerebrospinal fluid biomarkers specific to neuroinflammation (e.g., GFAP, NfL). It is important to note that our analyses controlled for age and included only those who were deemed cognitively stable; given the strong association between age and amyloid deposition level (*r* = 0.635, *p* < 0.001), our sample may have been underpowered to detect small‐sized associations, and further, these associations may be stronger in later AD disease stages (i.e., following MCI and dementia).

This study had several strengths and limitations. This study leveraged a large, multisite cohort of adults with DS and included a comprehensive panel of plasma inflammatory biomarkers, several of which are commonly used in clinical settings (e.g., CRP). Models simultaneously evaluated BMI, aging, and amyloid burden while adjusting for the impact of key covariates including biological sex, trisomy type, and data collection site. Sensitivity analyses were conducted to ensure findings were not driven by extreme values. Limitations include the cross‐sectional design, which precludes causal inferences, and limited diversity in race and ethnicity of the sample may constrain generalizability. BMI alone may not capture the complexity of metabolic dysfunction, especially in a population that has a high prevalence of obesity. Future research should incorporate additional metabolic indicators, such as insulin resistance, lipid profiles, and other markers of metabolic syndrome (blood glucose, waist circumference, adiponectin). The current study also did not have access to detailed information about the presence and severity of inflammatory‐related health conditions or medications. Future research should control for these conditions and medications that influence inflammation in the investigation of metaflammation and inflammaging.

Overall, these findings highlight the contribution of obesity, aging, and early amyloid deposition (i.e., preclinical AD stage) to systemic inflammation in adults with DS. Findings suggest that adults with DS who have BMI in the overweight or obesity range exhibit higher levels of systemic inflammation than those who are of normal weight. While early amyloid deposition showed limited associations with general inflammatory biomarkers, this underscores the potential need for neuroinflammation‐specific biomarkers to better capture amyloid‐driven inflammatory processes. The results emphasize the importance of targeted interventions to reduce systemic inflammation aimed at supporting healthy aging and metabolic health in DS. Future research and clinical trials should be deliberate in their selection of biomarkers that align with the biological processes of interest to optimize detection and intervention strategies.

## Funding

This research was funded by the National Institute on Aging (F31 AG085730; K01 AG083130; R01 AG070028; U01 AG051406; U01 AG051412; U19 AG068054). This study was supported in part by a core grant to the Waisman Center from the National Institute of Child Health and Human Development (P50 HD105353).

## Conflicts of Interest

The authors declare no conflicts of interest.

## Supporting information


**Figure S1:** Associations between BMI and markers of inflammation by biological sex. (a) beta‐2 macroglobulin (B2M; pg/mL), (b) C‐reactive protein (CRP). Lines represent linear mixed‐effects regression models adjusted for age, amyloid burden, biological sex, trisomy type, and site (random effect). *β* coefficients and FDR‐corrected *p* values (Benjamini and Hochberg [51]) are displayed within panels.

**Table S1:** Multilevel models of BMI, age, and amyloid predicting inflammation (excluding ±3 SD).

**Table S2:** Multilevel models of BMI, BMI × biological sex, age, and amyloid predicting inflammation.

**Table S3:** Multilevel models of BMI, BMI × biological sex, age, and amyloid predicting inflammation (excluding ±3 SD).

## Data Availability

The data that supports this study are available upon request in LONI (https://ida.loni.usc.edu/login.jsp).

## References

[1] G. de Graaf , F. Buckley , J. Dever , and B. G. Skotko , “Estimation of Live Birth and Population Prevalence of Down Syndrome in Nine U.S. States,” American Journal of Medical Genetics. Part A 173 (2017): 2710–2719. 28816027 10.1002/ajmg.a.38402

[2] E. B. Stallings , J. L. Isenburg , R. E. Rutkowski , et al., “National Population‐Based Estimates for Major Birth Defects, 2016–2020,” Birth Defects Research 116 (2024): e2301. 38277408 10.1002/bdr2.2301 PMC10898112

[3] D. Huggard , L. Kelly , E. Ryan , et al., “Increased Systemic Inflammation in Children With Down Syndrome,” Cytokine 127 (2020): 154938. 31785499 10.1016/j.cyto.2019.154938

[4] M. B. Trotta , J. B. Serro Azul , M. Wajngarten , S. G. Fonseca , A. C. Goldberg , and J. E. Kalil , “Inflammatory and Immunological Parameters in Adults With Down Syndrome,” Immunity and Ageing 8 (2011): 4. 21496308 10.1186/1742-4933-8-4 PMC3101128

[5] A. J. Bell and M. S. Bhate , “Prevalence of Overweight and Obesity in Down's Syndrome and Other Mentally Handicapped Adults Living in the Community,” Journal of Intellectual Disability Research 36, no. Pt 4 (1992): 359–364. 1388077 10.1111/j.1365-2788.1992.tb00534.x

[6] N. M. Oreskovic , N. T. Baumer , C. Di Camillo , et al., “Cardiometabolic Profiles in Children and Adults With Overweight and Obesity and Down Syndrome,” American Journal of Medical Genetics. Part A 191 (2023): 813–822. 36538912 10.1002/ajmg.a.63088

[7] R. J. Stancliffe , K. C. Lakin , S. A. Larson , et al., “Demographic Characteristics, Health Conditions, and Residential Service Use in Adults With Down Syndrome in 25 U.S. States,” Intellectual and Developmental Disabilities 50 (2012): 92–108. 22642964 10.1352/1934-9556-50.2.92

[8] D. Khanna , S. Khanna , P. Khanna , P. Kahar , and B. M. Patel , “Obesity: A Chronic Low‐Grade Inflammation and Its Markers,” Cureus 14, no. 2 (2022): e22711. 35386146 10.7759/cureus.22711 PMC8967417

[9] M. Bulló , P. García‐Lorda , I. Megias , and J. Salas‐Salvadó , “Systemic Inflammation, Adipose Tissue Tumor Necrosis Factor, and Leptin Expression,” Obesity Research 11 (2003): 525–531. 12690081 10.1038/oby.2003.74

[10] A. Festa , R. D'Agostino , K. Williams , et al., “The Relation of Body Fat Mass and Distribution to Markers of Chronic Inflammation,” International Journal of Obesity and Related Metabolic Disorders 25 (2001): 1407–1415. 11673759 10.1038/sj.ijo.0801792

[11] R. Mabrouk , H. Ghareeb , A. Shehab , et al., “Serum Visfatin, Resistin and IL‐18 in A Group of Egyptian Obese Diabetic and Non Diabetic Individuals,” Egyptian Journal of Immunology 20 (2013): 1–11. 23888552

[12] B. Glowinska , M. Urban , J. Peczynska , and B. Florys , “Soluble Adhesion Molecules (sICAM‐1, sVCAM‐1) and Selectins (sE Selectin, sP Selectin, sL Selectin) Levels in Children and Adolescents With Obesity, Hypertension, and Diabetes,” Metabolism 54 (2005): 1020–1026. 16092051 10.1016/j.metabol.2005.03.004

[13] M. Fructuoso , L. Rachdi , E. Philippe , et al., “Increased Levels of Inflammatory Plasma Markers and Obesity Risk in a Mouse Model of Down Syndrome,” Free Radical Biology and Medicine 114 (2018): 122–130. 28958596 10.1016/j.freeradbiomed.2017.09.021

[14] A. Gutierrez‐Hervas , S. Gómez‐Martínez , R. Izquierdo‐Gómez , et al., “Inflammation and Fatness in Adolescents With and Without Down Syndrome: UP & DOWN Study,” Journal of Intellectual Disability Research 64 (2020): 170–179. 31858639 10.1111/jir.12697

[15] M. Moreau , S. Benhaddou , R. Dard , et al., “Metabolic Diseases and Down Syndrome: How Are They Linked Together?,” Biomedicine 9 (2021): 221. 10.3390/biomedicines9020221 PMC7926648 33671490

[16] L. R. Chapman , I. V. P. Ramnarine , D. Zemke , A. Majid , and S. M. Bell , “Gene Expression Studies in Down Syndrome: What Do They Tell us About Disease Phenotypes?,” International Journal of Molecular Sciences 25 (2024): 2968. 38474215 10.3390/ijms25052968 PMC10932069

[17] B. B. Ganguly and N. N. Kadam , “Genetic Interactions and Co‐Operating Effects of Non‐HSA21 Genes on the Phenotypic Variability in Down Syndrome,” Gene Reports 38 (2025): 102106.

[18] J. A. Luchsinger , D. Pang , S. J. Krinsky‐McHale , et al., “Obesity, Diabetes and Their Metabolic Correlates in Middle‐Aged Adults With Down Syndrome,” Journal of Intellectual Disability Research 68 (2024): 212–222. 37899501 10.1111/jir.13103 PMC10872834

[19] K. L. Brugge , G. L. Grove , P. Clopton , M. J. Grove , and D. J. Piacquadio , “Evidence for Accelerated Skin Wrinkling Among Developmentally Delayed Individuals With Down's Syndrome,” Mechanisms of Ageing and Development 70 (1993): 213–225. 8246635 10.1016/0047-6374(93)90049-w

[20] R. Sureshbabu , R. Kumari , S. Ranugha , R. Sathyamoorthy , C. Udayashankar , and P. Oudeacoumar , “Phenotypic and Dermatological Manifestations in Down Syndrome,” Dermatology Online Journal 17 (2011): 3. 21382286

[21] N. Congdon , J. R. Vingerling , B. E. K. Klein , et al., “Prevalence of Cataract and Pseudophakia/Aphakia Among Adults in the United States,” Archives of Ophthalmology 2004, no. 122 (1960): 487–494. 10.1001/archopht.122.4.487 15078665

[22] S. J. Krinsky‐McHale , E. C. Jenkins , W. B. Zigman , and W. Silverman , “Ophthalmic Disorders in Adults With Down Syndrome,” Current Gerontology and Geriatrics Research 2012 (2012): 974253. 22570648 10.1155/2012/974253 PMC3337581

[23] J. Carr and S. Hollins , “Menopause in Women With Learning Disabilities,” Journal of Intellectual Disability Research 39, no. Pt 2 (1995): 137–139. 7787383 10.1111/j.1365-2788.1995.tb00481.x

[24] N. Schupf , W. Zigman , D. Kapell , J. H. Lee , J. Kline , and B. Levin , “Early Menopause in Women With Down's Syndrome,” Journal of Intellectual Disability Research 41, no. Pt 3 (1997): 264–267. 9219076 10.1046/j.1365-2788.1997.03838.x

[25] M. K. Kim , K.‐J. Yun , H. J. Chun , et al., “Clinical Utility of Serum Beta‐2‐Microglobulin as a Predictor of Diabetic Complications in Patients With Type 2 Diabetes Without Renal Impairment,” Diabetes and Metabolism 40 (2014): 459–465. 25303803 10.1016/j.diabet.2014.08.002

[26] L. Tang , M. Liu , Y. Tao , et al., “Association of Aging Acceleration With Serum Neurofilament Light Chain Levels: Implications for the Roles of Modifiable Aging Factors,” Journal of Affective Disorders 372 (2025): 481–490. 39638062 10.1016/j.jad.2024.12.023

[27] C. Franceschi and J. Campisi , “Chronic Inflammation (Inflammaging) and Its Potential Contribution to Age‐Associated Diseases,” Journals of Gerontology. Series A, Biological Sciences and Medican Sciences 69, no. S1 (2014): S4–S9. 10.1093/gerona/glu057 24833586

[28] J. Fortea , S. H. Zaman , S. Hartley , M. S. Rafii , E. Head , and M. Carmona‐Iragui , “Alzheimer's Disease Associated With Down Syndrome: A Genetic Form of Dementia,” Lancet Neurology 20 (2021): 930–942. 34687637 10.1016/S1474-4422(21)00245-3 PMC9387748

[29] M. McCarron , P. McCallion , E. Reilly , P. Dunne , R. Carroll , and N. Mulryan , “A Prospective 20‐Year Longitudinal Follow‐Up of Dementia in Persons With Down Syndrome,” Journal of Intellectual Disability Research 61 (2017): 843–852. 28664561 10.1111/jir.12390

[30] M. S. Rafii , B. M. Ances , N. Schupf , et al., “The AT(N) Framework for Alzheimer's Disease in Adults With Down Syndrome,” Alzheimer's and Dementia (Amst) 12 (2020): e12062. 10.1002/dad2.12062 PMC7588820 33134477

[31] M. F. Iulita , D. Garzón Chavez , M. Klitgaard Christensen , et al., “Association of Alzheimer Disease With Life Expectancy in People With Down Syndrome,” JAMA Network Open 5 (2022): e2212910. 35604690 10.1001/jamanetworkopen.2022.12910 PMC9127560

[32] E. Doran , D. Keator , E. Head , et al., “Down Syndrome, Partial Trisomy 21, and Absence of Alzheimer's Disease: The Role of APP,” Journal of Alzheimer's Disease 56 (2017): 459–470. 10.3233/JAD-160836 PMC5662115 27983553

[33] B. M. Bettcher , J. Neuhaus , M. J. Wynn , et al., “Increases in a Pro‐Inflammatory Chemokine, MCP‐1, Are Related to Decreases in Memory Over Time,” Frontiers in Aging Neuroscience 11 (2019): 11. 30814948 10.3389/fnagi.2019.00025 PMC6381047

[34] S. Bradburn , C. Murgatroyd , and N. Ray , “Neuroinflammation in Mild Cognitive Impairment and Alzheimer's Disease: A Meta‐Analysis,” Ageing Research Reviews 50 (2019): 1–8. 30610927 10.1016/j.arr.2019.01.002

[35] N. Mattsson , U. Andreasson , H. Zetterberg , and K. Blennow , “For the Alzheimer's Disease Neuroimaging Initiative. Association of Plasma Neurofilament Light With Neurodegeneration in Patients With Alzheimer Disease,” JAMA Neurology 74 (2017): 557–566. 28346578 10.1001/jamaneurol.2016.6117 PMC5822204

[36] M. T. Heneka and M. K. O'Banion , “Inflammatory Processes in Alzheimer's Disease,” Journal of Neuroimmunology 184 (2007): 69–91. 17222916 10.1016/j.jneuroim.2006.11.017

[37] B. Twarowski and M. Herbet , “Inflammatory Processes in Alzheimer's Disease—Pathomechanism, Diagnosis and Treatment: A Review,” International Journal of Molecular Sciences 24 (2023): 6518. 37047492 10.3390/ijms24076518 PMC10095343

[38] M. F. Iulita , A. Ower , C. Barone , et al., “An Inflammatory and Trophic Disconnect Biomarker Profile Revealed in Down Syndrome Plasma: Relation to Cognitive Decline and Longitudinal Evaluation,” Alzheimer's and Dementia 12 (2016): 1132–1148. 10.1016/j.jalz.2016.05.001 27452424

[39] M. E. Petersen , L. Flores‐Aguilar , E. Head , et al., “Blood Biomarkers in Down Syndrome: Facilitating Alzheimer's Disease Detection and Monitoring,” Alzheimer's and Dementia 21 (2025): e14364. 10.1002/alz.14364 PMC11782192 39535517

[40] R. Varadhan , W. Yao , A. Matteini , et al., “Simple Biologically Informed Inflammatory Index of Two Serum Cytokines Predicts 10 Year All‐Cause Mortality in Older Adults,” Journals of Gerontology. Series A, Biological Sciences and Medical Sciences 69 (2014): 165–173. 23689826 10.1093/gerona/glt023 PMC4038244

[41] A. Wikby , B.‐O. Nilsson , R. Forsey , et al., “The Immune Risk Phenotype Is Associated With IL‐6 in the Terminal Decline Stage: Findings From the Swedish NONA Immune Longitudinal Study of Very Late Life Functioning,” Mechanisms of Ageing and Development 127 (2006): 695–704. 16750842 10.1016/j.mad.2006.04.003

[42] B. L. Handen , M. Mapstone , S. Hartley , et al., “The Alzheimer's Biomarker Consortium‐Down Syndrome (ABC‐DS): A 10‐Year Report,” Alzheimer's and Dementia 21 (2025): e70294. 10.1002/alz.70294 PMC12079517 40371686

[43] G. H. Roid and M. Pomplun , “The Stanford‐Binet Intelligence Scales, Fifth Edition,” in Contemporary Intellectual Assessment: Theories, Tests, and Issues, 3rd ed., eds. D. P. Flanagan and P. L. Harrison (Guilford Press, 2012), 249–268.

[44] A. S. Kaufman and N. L. Kaufman , Kaufman Brief Intelligence Test, 2nd ed. (Pearson Assessments, 2004).

[45] CDC , “Adult BMI Categories,” updated March 19, 2024, https://www.cdc.gov/bmi/adult‐calculator/bmi‐categories.html.

[46] S. E. O'Bryant , G. Xiao , F. Zhang , et al., “Validation of a Serum Screen for Alzheimer's Disease Across Assay Platforms, Species, and Tissues,” Journal of Alzheimer's Disease 42 (2014): 1325–1335. 10.3233/JAD-141041 PMC4400808 25024345

[47] S. E. O'Bryant , M. Edwards , L. Johnson , et al., “A Blood Screening Test for Alzheimer's Disease,” Alzheimer's and Dementia (Amst) 3 (2016): 83–90. 10.1016/j.dadm.2016.06.004 PMC4941038 27453929

[48] W. E. Klunk , R. A. Koeppe , J. C. Price , et al., “The Centiloid Project: Standardizing Quantitative Amyloid Plaque Estimation by PET,” Alzheimer's and Dementia 11, no. 1 (2015): 1–15.e1–e4. 10.1016/j.jalz.2014.07.003 PMC4300247 25443857

[49] W. Luo , D. S. Minhas , E. D. Rubenstein , et al., “Development and Evaluation of Image Preprocessing Pipelines for the Centiloid Method on Down Syndrome Data,” Alzheimer's and Dementia 21 (2025): e70712. 10.1002/alz.70712 PMC12514937 41074907

[50] D. Bates , M. Mächler , B. Bolker , and S. Walker , “Fitting Linear Mixed‐Effects Models Using lme4,” Journal of Statistical Software 67, no. 1 (2015): 1–48, 10.18637/jss.v067.i01.

[51] Y. Benjamini and Y. Hochberg , “Controlling the False Discovery Rate: A Practical and Powerful Approach to Multiple Testing,” Journal of the Royal Statistical Society. Series B, Statistical Methodology 57 (1995): 289–300.

[52] E. Chang , M. Varghese , and K. Singer , “Gender and Sex Differences in Adipose Tissue,” Current Diabetes Reports 18 (2018): 69. 30058013 10.1007/s11892-018-1031-3 PMC6525964

[53] A. A. Kammerlander , A. Lyass , T. F. Mahoney , et al., “Sex Differences in the Associations of Visceral Adipose Tissue and Cardiometabolic and Cardiovascular Disease Risk: The Framingham Heart Study,” Journal of the American Heart Association 10 (2021): e019968. 33998254 10.1161/JAHA.120.019968 PMC8483556

[54] H. S. Park , J. Y. Park , and R. Yu , “Relationship of Obesity and Visceral Adiposity With Serum Concentrations of CRP, TNF‐α and IL‐6,” Diabetes Research and Clinical Practice 69 (2005): 29–35. 15955385 10.1016/j.diabres.2004.11.007

[55] F. M. Schmidt , J. Weschenfelder , C. Sander , et al., “Inflammatory Cytokines in General and Central Obesity and Modulating Effects of Physical Activity,” PLoS One 10 (2015): e0121971. 25781614 10.1371/journal.pone.0121971 PMC4363366

[56] L. T. Ptomey and W. Wittenbrook , “Position of the Academy of Nutrition and Dietetics: Nutrition Services for Individuals With Intellectual and Developmental Disabilities and Special Health Care Needs,” Journal of the Academy of Nutrition and Dietetics 115 (2015): 593–608. 25819518 10.1016/j.jand.2015.02.002

[57] I.‐S. Shin and E.‐Y. Park , “Meta‐Analysis of the Effect of Exercise Programs for Individuals With Intellectual Disabilities,” Research in Developmental Disabilities 33 (2012): 1937–1947. 22728605 10.1016/j.ridd.2012.05.019

[58] A. M. Abbatecola , A. Giuliani , L. Biscetti , et al., “Circulating Biomarkers of Inflammaging and Alzheimer's Disease to Track Age‐Related Trajectories of Dementia: Can We Develop a Clinically Relevant Composite Combination?,” Ageing Research Reviews 96 (2024): 102257. 38437884 10.1016/j.arr.2024.102257

[59] C. Annweiler , R. Bataille , N. Ferrière , D. Douillet , B. Fantino , and O. Beauchet , “Plasma Beta‐2 Microglobulin as a Marker of Frailty in Older Adults: A Pilot Study,” Journals of Gerontology. Series A, Biological Sciences and Medical Sciences 66 (2011): 1077–1079. 21743090 10.1093/gerona/glr104

[60] R. Dominici , D. Finazzi , L. Polito , et al., “Comparison of β2‐Microglobulin Serum Level Between Alzheimer's Patients, Cognitive Healthy and Mild Cognitive Impaired Individuals,” Biomarkers 23 (2018): 603–608. 29741401 10.1080/1354750X.2018.1468825

[61] M. Mahdavi , S. Karima , S. Rajaei , et al., “Plasma Cytokines Profile in Subjects With Alzheimer's Disease: Interleukin 1 Alpha as a Candidate for Target Therapy,” Galen Medical Journal 10 (2021): e1974. 35434157 10.31661/gmj.v10i0.1974 PMC9007609

