# Supplementary material for: Inflammation Associated With Obesity, Aging, and Amyloid Burden in Adults With Down Syndrome
Source: Obesity (Silver Spring). 2026 Jun 5;34(7):1457–67. doi: 10.1002/oby.70229 (PMC13306135; doi:10.1002/oby.70229)
Supplement: Supplementary file 1 — Figure S1: Associations between BMI and markers of inflammation by biological sex. (a) beta‐2 macroglobulin (B2M; pg/mL), (b) C‐reactive protein (CRP). Lines represent linear mixed‐effects regression models adjusted for age, amyloid burden, biological sex, trisomy type, and site (random effect). β coefficients and FDR‐corrected p values (Benjamini and Hochberg [51]) are displayed within panels. [file OBY-34-1457-s001.docx]

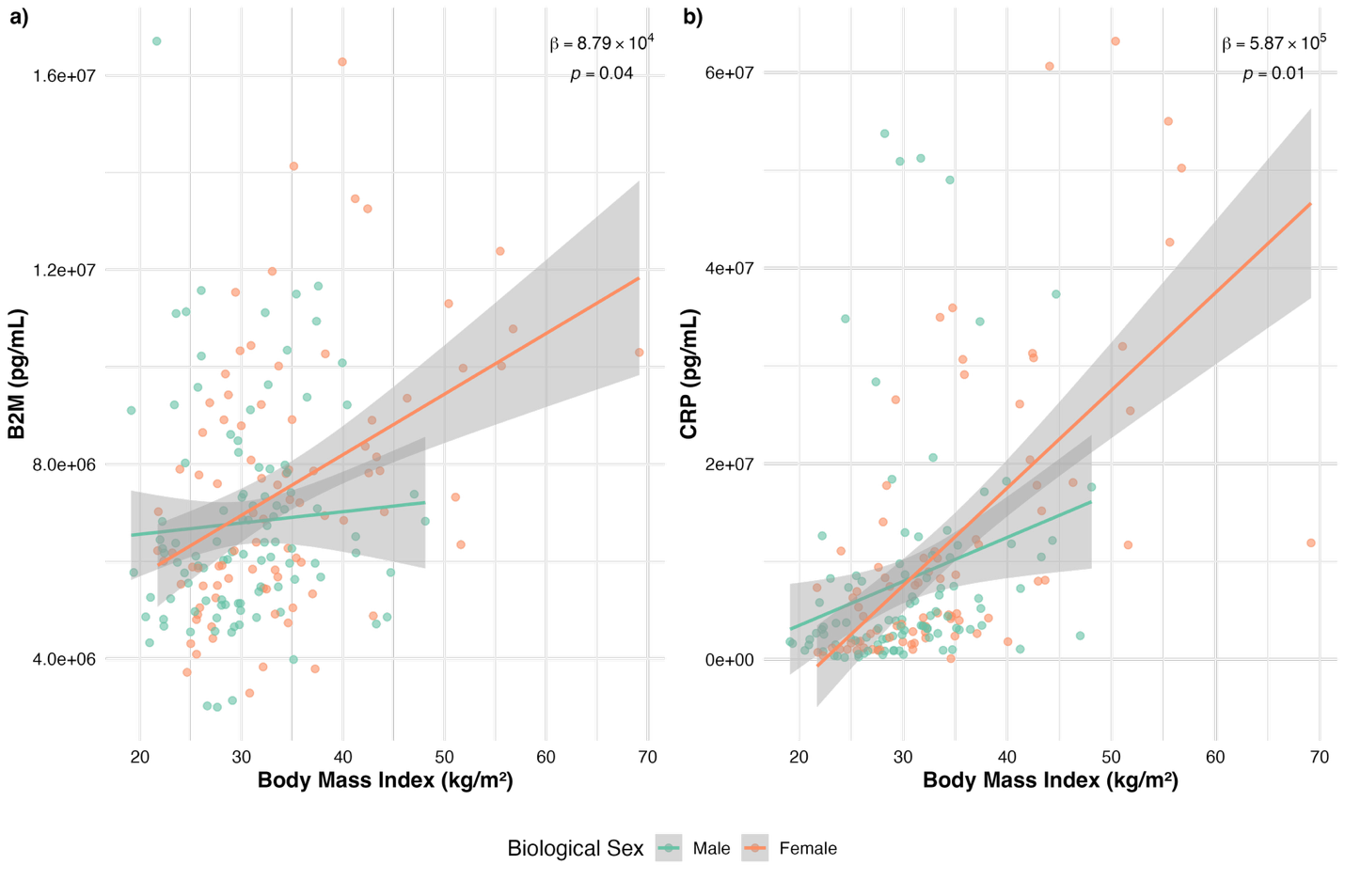


**Figure S1. Associations between body mass index (BMI) and markers of inflammation by biological sex.** (a) beta-2 macroglobulin (B2M; pg/mL), b) C-reactive Protein (CRP). Lines represent linear mixed-effects regression models adjusted for age ,amyloid burden, biological sex, trisomy type, and site (random effect). β coefficients and FDR-corrected p-values (Benjamini & Hochberg, 1995) are displayed within panels.
