# Supplementary material for: Inflammation Associated With Obesity, Aging, and Amyloid Burden in Adults With Down Syndrome
Source: Obesity (Silver Spring). 2026 Jun 5;34(7):1457–67. doi: 10.1002/oby.70229 (PMC13306135; doi:10.1002/oby.70229)
Supplement: Supplementary file 2 — Table S1: Multilevel models of BMI, age, and amyloid predicting inflammation (excluding ±3 SD). [file OBY-34-1457-s002.docx]

Table S1. Multilevel Models of Body Mass Index, Age, and Amyloid Predicting Inflammation (excluding ±3 standard deviations)

| Outcome | Fixed Effects | B | SE | 95% CI | t | *p* |
| --- | --- | --- | --- | --- | --- | --- |
| **CRP** | **Fixed Effects** |  |  |  |  |  |
|  | Intercept | -2.74e+07 | 6.60e+06 | [-4.04e+07, -1.45e+07] | -4.16 | <0.001 |
|  | BMI | 7.96e+05 | 1.12e+05 | [5.77e+05, 1.02e+06] | 7.12 | <0.001 |
|  | Age | 1.84e+05 | 1.23e+05 | [-56786.839, 4.24e+05] | 1.50 | 0.135 |
|  | Centiloid | -13337.842 | 36599.081 | [-85072.042, 58396.357] | -0.36 | 0.716 |
|  | Biological Sex (Female) | 9.08e+05 | 1.76e+06 | [-2.54e+06, 4.36e+06] | 0.52 | 0.606 |
|  | Trisomy Type (Full Trisomy) | 2.57e+06 | 1.66e+06 | [-6.91e+05, 5.83e+06] | 1.54 | 0.122 |
| **IL-6** | **Fixed Effects** |  |  |  |  |  |
|  | Intercept | -0.993 | 0.344 | [-1.667, -0.319] | -2.89 | 0.004 |
|  | BMI | 0.034 | 0.006 | [0.023, 0.046] | 5.85 | <0.001 |
|  | Age | 0.015 | 0.006 | [0.002, 0.027] | 2.30 | 0.023 |
|  | Centiloid | 0.001 | 0.002 | [-0.003, 0.005] | 0.41 | 0.680 |
|  | Biological Sex (Female) | 0.084 | 0.091 | [-0.094, 0.261] | 0.93 | 0.355 |
|  | Trisomy Type (Full Trisomy) | 0.191 | 0.084 | [0.026, 0.355] | 2.27 | 0.024 |
| **IL-10** | **Fixed Effects** |  |  |  |  |  |
|  | Intercept | 0.641 | 0.200 | [0.249, 1.033] | 3.21 | 0.002 |
|  | BMI | 0.000 | 0.003 | [-0.006, 0.007] | 0.07 | 0.942 |
|  | Age | -0.000 | 0.004 | [-0.008, 0.007] | -0.12 | 0.904 |
|  | Centiloid | -0.002 | 0.001 | [-0.004, 0.001] | -1.42 | 0.158 |
|  | Biological Sex (Female) | 0.011 | 0.053 | [-0.094, 0.115] | 0.20 | 0.841 |
|  | Trisomy Type (Full Trisomy) | -0.059 | 0.049 | [-0.155, 0.038] | -1.19 | 0.236 |
| **TNF-α** | **Fixed Effects** |  |  |  |  |  |
|  | Intercept | 1.590 | 0.410 | [0.787, 2.394] | 3.88 | <0.001 |
|  | BMI | 0.025 | 0.007 | [0.011, 0.038] | 3.53 | <0.001 |
|  | Age | 0.014 | 0.008 | [-0.001, 0.029] | 1.86 | 0.065 |
|  | Centiloid | -0.002 | 0.002 | [-0.006, 0.003] | -0.84 | 0.401 |
|  | Biological Sex (Female) | -0.093 | 0.109 | [-0.306, 0.120] | -0.86 | 0.394 |
|  | Trisomy Type (Full Trisomy) | 0.160 | 0.097 | [-0.031, 0.351] | 1.64 | 0.102 |
| **A2M** | **Fixed Effects** |  |  |  |  |  |
|  | Intercept | 6.96e+08 | 1.51e+08 | [3.99e+08, 9.92e+08] | 4.60 | <0.001 |
|  | BMI | -1.07e+06 | 2.48e+06 | [-5.92e+06, 3.79e+06] | -0.43 | 0.666 |
|  | Age | 3.86e+06 | 2.81e+06 | [-1.64e+06, 9.37e+06] | 1.37 | 0.169 |
|  | Centiloid | -8.98e+05 | 8.13e+05 | [-2.49e+06, 6.95e+05] | -1.11 | 0.269 |
|  | Biological Sex (Female) | 1.48e+08 | 3.81e+07 | [7.39e+07, 2.23e+08] | 3.90 | <0.001 |
|  | Trisomy Type (Full Trisomy) | 4.90e+07 | 3.39e+07 | [-1.75e+07, 1.15e+08] | 1.45 | 0.148 |
| **B2M** | **Fixed Effects** |  |  |  |  |  |
|  | Intercept | -1.22e+06 | 1.11e+06 | [-3.40e+06, 9.53e+05] | -1.10 | 0.271 |
|  | BMI | 1.06e+05 | 18816.027 | [68759.725, 1.43e+05] | 5.61 | <0.001 |
|  | Age | 1.09e+05 | 20822.744 | [68554.522, 1.50e+05] | 5.25 | <0.001 |
|  | Centiloid | -5299.880 | 6163.811 | [-17380.950, 6781.190] | -0.86 | 0.390 |
|  | Biological Sex (Female) | 3.59e+05 | 2.92e+05 | [-2.14e+05, 9.32e+05] | 1.23 | 0.220 |
|  | Trisomy Type (Full Trisomy) | -1493.960 | 2.72e+05 | [-5.34e+05, 5.31e+05] | -0.01 | 0.996 |
| **IL-18** | **Fixed Effects** |  |  |  |  |  |
|  | Intercept | 53.059 | 23.188 | [7.611, 98.508] | 2.29 | 0.023 |
|  | BMI | 1.567 | 0.393 | [0.796, 2.337] | 3.99 | <0.001 |
|  | Age | -0.037 | 0.431 | [-0.881, 0.808] | -0.09 | 0.932 |
|  | Centiloid | 0.162 | 0.129 | [-0.091, 0.414] | 1.26 | 0.211 |
|  | Biological Sex (Female) | -2.648 | 6.126 | [-14.655, 9.360] | -0.43 | 0.666 |
|  | Trisomy Type (Full Trisomy) | 2.200 | 5.524 | [-8.627, 13.028] | 0.40 | 0.691 |
| **sICAM-1** | **Fixed Effects** |  |  |  |  |  |
|  | Intercept | 1.74e+05 | 53973.041 | [68469.512, 2.80e+05] | 3.23 | 0.002 |
|  | BMI | 3331.667 | 932.632 | [1503.709, 5159.625] | 3.57 | <0.001 |
|  | Age | 1702.086 | 999.123 | [-256.194, 3660.367] | 1.70 | 0.091 |
|  | Centiloid | -427.272 | 298.558 | [-1012.446, 157.902] | -1.43 | 0.154 |
|  | Biological Sex (Female) | 9308.643 | 14198.113 | [-18519.658, 37136.945] | 0.66 | 0.513 |
|  | Trisomy Type (Full Trisomy) | -551.859 | 12780.893 | [-25602.409, 24498.692] | -0.04 | 0.966 |

Note. B=unstandardized regression coefficient; SE=standard error; CI=confidence interval. Large values are displayed in scientific notation for readability. All models corrected for multiple comparisons using the false discovery rate (Benjamini–Hochberg, 1995). Sex reference = Male; site included as a random intercept. BMI=Body Mass Index; CRP=C-reactive Protein; IL=interleukin; TNF- α=Tumor Necrosis Factor–Alpha; A2M=alpha-2 macroglobulin; B2m=beta-2 macroglobulin; sICAM-1=soluble intercellular adhesion molecule-1.
