# Supplementary material for: Inflammation Associated With Obesity, Aging, and Amyloid Burden in Adults With Down Syndrome
Source: Obesity (Silver Spring). 2026 Jun 5;34(7):1457–67. doi: 10.1002/oby.70229 (PMC13306135; doi:10.1002/oby.70229)
Supplement: Supplementary file 3 — Table S2: Multilevel models of BMI, BMI × biological sex, age, and amyloid predicting inflammation. [file OBY-34-1457-s004.docx]

Table S2. Multilevel Models of Body Mass Index, BMI x Biological Sex, Age, and Amyloid Predicting Inflammation

| Outcome | Fixed Effects | B | SE | 95% CI | t | *p* |
| --- | --- | --- | --- | --- | --- | --- |
| **CRP** | **Fixed Effects** |  |  |  |  |  |
|  | Intercept | -3.93e+06 | 1.97e+07 | [-4.26e+07, 3.47e+07] | -0.20 | 0.842 |
|  | BMI | -1.78e+05 | 5.77e+05 | [-1.31e+06, 9.53e+05] | -0.31 | 0.757 |
|  | Biological Sex (Female) | -1.35e+07 | 1.09e+07 | [-3.48e+07, 7.78e+06] | -1.24 | 0.213 |
|  | Age | 2.23e+05 | 1.76e+05 | [-1.22e+05, 5.68e+05] | 1.27 | 0.205 |
|  | Centiloid | -47663.204 | 52554.968 | [-1.51e+05, 55344.534] | -0.91 | 0.365 |
|  | Trisomy Type (Full Trisomy) | 6.40e+06 | 2.26e+06 | [1.98e+06, 1.08e+07] | 2.84 | 0.005 |
|  | BMI x Biological Sex | 5.41e+05 | 3.34e+05 | [-1.13e+05, 1.20e+06] | 1.62 | 0.105 |
| **IL-6** | **Fixed Effects** |  |  |  |  |  |
|  | Intercept | -0.350 | 0.927 | [-2.167, 1.466] | -0.38 | 0.706 |
|  | BMI | 0.013 | 0.027 | [-0.040, 0.067] | 0.48 | 0.628 |
|  | Biological Sex (Female) | -0.301 | 0.512 | [-1.304, 0.703] | -0.59 | 0.558 |
|  | Age | 0.011 | 0.008 | [-0.005, 0.027] | 1.30 | 0.196 |
|  | Centiloid | 0.005 | 0.002 | [0.000, 0.010] | 2.07 | 0.040 |
|  | Trisomy Type (Full Trisomy) | 0.246 | 0.106 | [0.037, 0.454] | 2.31 | 0.022 |
|  | BMI x Biological Sex | 0.014 | 0.016 | [-0.016, 0.045] | 0.92 | 0.361 |
| **IL-10** | **Fixed Effects** |  |  |  |  |  |
|  | Intercept | 0.650 | 0.596 | [-0.519, 1.818] | 1.09 | 0.278 |
|  | BMI | -0.005 | 0.018 | [-0.039, 0.029] | -0.29 | 0.770 |
|  | Biological Sex (Female) | 0.117 | 0.330 | [-0.529, 0.763] | 0.35 | 0.723 |
|  | Age | -0.000 | 0.005 | [-0.011, 0.010] | -0.07 | 0.944 |
|  | Centiloid | -0.002 | 0.002 | [-0.005, 0.002] | -0.95 | 0.343 |
|  | Trisomy Type (Full Trisomy) | 0.033 | 0.068 | [-0.101, 0.168] | 0.49 | 0.626 |
|  | BMI x Biological Sex | -0.001 | 0.010 | [-0.021, 0.019] | -0.12 | 0.906 |
| **TNF-α** | **Fixed Effects** |  |  |  |  |  |
|  | Intercept | 1.321 | 1.064 | [-0.764, 3.406] | 1.24 | 0.216 |
|  | BMI | 0.030 | 0.031 | [-0.031, 0.090] | 0.96 | 0.339 |
|  | Biological Sex (Female) | 0.131 | 0.583 | [-1.012, 1.275] | 0.23 | 0.822 |
|  | Age | 0.015 | 0.010 | [-0.004, 0.033] | 1.51 | 0.132 |
|  | Centiloid | -0.003 | 0.003 | [-0.008, 0.003] | -0.98 | 0.327 |
|  | Trisomy Type (Full Trisomy) | 0.099 | 0.121 | [-0.139, 0.337] | 0.82 | 0.415 |
|  | BMI x Biological Sex | -0.002 | 0.018 | [-0.037, 0.033] | -0.09 | 0.924 |
| **A2M** | **Fixed Effects** |  |  |  |  |  |
|  | Intercept | 9.68e+08 | 3.36e+08 | [3.10e+08, 1.63e+09] | 2.88 | 0.004 |
|  | BMI | -1.07e+07 | 9.61e+06 | [-2.96e+07, 8.11e+06] | -1.12 | 0.264 |
|  | Biological Sex (Female) | 1.32e+06 | 1.81e+08 | [-3.54e+08, 3.57e+08] | 0.01 | 0.994 |
|  | Age | 5.69e+06 | 3.09e+06 | [-3.60e+05, 1.17e+07] | 1.84 | 0.065 |
|  | Centiloid | -1.43e+06 | 8.96e+05 | [-3.18e+06, 3.29e+05] | -1.59 | 0.111 |
|  | Trisomy Type (Full Trisomy) | 5.16e+07 | 3.77e+07 | [-2.24e+07, 1.26e+08] | 1.37 | 0.172 |
|  | BMI x Biological Sex | 4.59e+06 | 5.58e+06 | [-6.34e+06, 1.55e+07] | 0.82 | 0.411 |
| **B2M** | **Fixed Effects** |  |  |  |  |  |
|  | Intercept | 2.59e+06 | 2.52e+06 | [-2.34e+06, 7.53e+06] | 1.03 | 0.303 |
|  | BMI | -43960.053 | 73951.225 | [-1.89e+05, 1.01e+05] | -0.59 | 0.552 |
|  | Biological Sex (Female) | -2.37e+06 | 1.39e+06 | [-5.10e+06, 3.61e+05] | -1.70 | 0.089 |
|  | Age | 1.21e+05 | 22331.765 | [77111.879, 1.65e+05] | 5.41 | <0.001 |
|  | Centiloid | -6461.006 | 6701.889 | [-19596.709, 6674.697] | -0.96 | 0.335 |
|  | Trisomy Type (Full Trisomy) | 3.52e+05 | 2.89e+05 | [-2.15e+05, 9.18e+05] | 1.22 | 0.224 |
|  | BMI x Biological Sex | 8.790e+04 | 42758.318 | [4089.431, 1.72e+05] | 2.06 | 0.040 |
| **IL-18** | **Fixed Effects** |  |  |  |  |  |
|  | Intercept | 92.032 | 51.035 | [-7.995, 192.060] | 1.80 | 0.073 |
|  | BMI | 0.029 | 1.498 | [-2.907, 2.965] | 0.02 | 0.985 |
|  | Biological Sex (Female) | -30.729 | 28.199 | [-85.998, 24.541] | -1.09 | 0.277 |
|  | Age | 0.116 | 0.452 | [-0.770, 1.003] | 0.26 | 0.797 |
|  | Centiloid | 0.150 | 0.136 | [-0.116, 0.416] | 1.11 | 0.270 |
|  | Trisomy Type (Full Trisomy) | 2.238 | 5.856 | [-9.240, 13.716] | 0.38 | 0.703 |
|  | BMI x Biological Sex | 0.980 | 0.866 | [-0.718, 2.677] | 1.13 | 0.260 |
| **sICAM-1** | **Fixed Effects** |  |  |  |  |  |
|  | Intercept | 3.06e+05 | 1.24e+05 | [63430.783, 5.49e+05] | 2.47 | 0.014 |
|  | BMI | -1343.598 | 3618.789 | [-8436.424, 5749.229] | -0.37 | 0.711 |
|  | Biological Sex (Female) | -79686.464 | 68138.214 | [-2.13e+05, 53864.435] | -1.17 | 0.244 |
|  | Age | 1823.675 | 1112.302 | [-356.437, 4003.787] | 1.64 | 0.103 |
|  | Centiloid | -493.763 | 331.048 | [-1142.617, 155.090] | -1.49 | 0.138 |
|  | Trisomy Type (Full Trisomy) | -2261.626 | 14164.272 | [-30023.600, 25500.348] | -0.16 | 0.873 |
|  | BMI x Biological Sex | 3141.752 | 2093.606 | [-961.715, 7245.219] | 1.50 | 0.135 |

Note. B=unstandardized regression coefficient; SE=standard error; CI=confidence interval. Large values are displayed in scientific notation for readability. All models corrected for multiple comparisons using the false discovery rate (Benjamini–Hochberg, 1995). Sex reference = Male; site included as a random intercept. BMI=Body Mass Index; CRP=C-reactive Protein; IL=interleukin; TNF- α=Tumor Necrosis Factor–Alpha; A2M=alpha-2 macroglobulin; B2M=beta-2 macroglobulin; sICAM-1=soluble intercellular adhesion molecule-1.
