# Supplementary material for: Inflammation Associated With Obesity, Aging, and Amyloid Burden in Adults With Down Syndrome
Source: Obesity (Silver Spring). 2026 Jun 5;34(7):1457–67. doi: 10.1002/oby.70229 (PMC13306135; doi:10.1002/oby.70229)
Supplement: Supplementary file 4 — Table S3: Multilevel models of BMI, BMI × biological sex, age, and amyloid predicting inflammation (excluding ±3 SD). [file OBY-34-1457-s003.docx]

Table S3. Multilevel Models of Body Mass Index, BMI x Biological Sex, Age, and Amyloid Predicting Inflammation (excluding ±3 standard deviations)

| Outcome | Fixed Effects | B | SE | 95% CI | t | *p* |
| --- | --- | --- | --- | --- | --- | --- |
| **CRP** | **Fixed Effects** |  |  |  |  |  |
|  | Intercept | 2.98e+06 | 1.35e+07 | [-2.35e+07, 2.94e+07] | 0.22 | 0.825 |
|  | BMI | -1.78e+05 | 3.94e+05 | [-9.51e+05, 5.95e+05] | -0.45 | 0.651 |
|  | Biological Sex (Female) | -1.77e+07 | 7.44e+06 | [-3.23e+07, -3.11e+06] | -2.38 | 0.017 |
|  | Age | 1.70e+05 | 1.21e+05 | [-68101.489, 4.07e+05] | 1.40 | 0.163 |
|  | Centiloid | -17013.136 | 36089.704 | [-87748.956, 53722.684] | -0.47 | 0.637 |
|  | Trisomy Type (Full Trisomy) | 3.03e+06 | 1.65e+06 | [-1.94e+05, 6.26e+06] | 1.84 | 0.065 |
|  | BMI x Biological Sex | 5.87e+05 | 2.28e+05 | [1.40e+05, 1.04e+06] | 2.57 | 0.010 |
| **IL-6** | **Fixed Effects** |  |  |  |  |  |
|  | Intercept | -0.730 | 0.717 | [-2.136, 0.676] | -1.02 | 0.310 |
|  | BMI | 0.026 | 0.021 | [-0.015, 0.067] | 1.23 | 0.221 |
|  | Biological Sex (Female) | -0.080 | 0.402 | [-0.867, 0.707] | -0.20 | 0.843 |
|  | Age | 0.015 | 0.006 | [0.002, 0.027] | 2.28 | 0.024 |
|  | Centiloid | 0.001 | 0.002 | [-0.003, 0.005] | 0.41 | 0.686 |
|  | Trisomy Type (Full Trisomy) | 0.196 | 0.085 | [0.029, 0.362] | 2.31 | 0.022 |
|  | BMI x Biological Sex | 0.005 | 0.012 | [-0.019, 0.029] | 0.42 | 0.677 |
| **IL-10** | **Fixed Effects** |  |  |  |  |  |
|  | Intercept | 0.766 | 0.420 | [-0.057, 1.589] | 1.82 | 0.070 |
|  | BMI | -0.004 | 0.012 | [-0.028, 0.020] | -0.30 | 0.761 |
|  | Biological Sex (Female) | -0.066 | 0.233 | [-0.524, 0.391] | -0.28 | 0.777 |
|  | Age | -0.000 | 0.004 | [-0.008, 0.007] | -0.13 | 0.898 |
|  | Centiloid | -0.002 | 0.001 | [-0.004, 0.001] | -1.43 | 0.156 |
|  | Trisomy Type (Full Trisomy) | -0.057 | 0.050 | [-0.155, 0.040] | -1.15 | 0.252 |
|  | BMI x Biological Sex | 0.002 | 0.007 | [-0.012, 0.016] | 0.34 | 0.736 |
| **TNF-α** | **Fixed Effects** |  |  |  |  |  |
|  | Intercept | 1.337 | 0.855 | [-0.340, 3.013] | 1.56 | 0.120 |
|  | BMI | 0.033 | 0.025 | [-0.016, 0.082] | 1.31 | 0.192 |
|  | Biological Sex (Female) | 0.063 | 0.474 | [-0.867, 0.992] | 0.13 | 0.895 |
|  | Age | 0.014 | 0.008 | [-0.001, 0.029] | 1.87 | 0.064 |
|  | Centiloid | -0.002 | 0.002 | [-0.006, 0.003] | -0.83 | 0.410 |
|  | Trisomy Type (Full Trisomy) | 0.156 | 0.098 | [-0.036, 0.349] | 1.59 | 0.113 |
|  | BMI x Biological Sex | -0.005 | 0.015 | [-0.033, 0.024] | -0.34 | 0.736 |
| **A2M** | **Fixed Effects** |  |  |  |  |  |
|  | Intercept | 8.35e+08 | 3.05e+08 | [2.37e+08, 1.43e+09] | 2.74 | 0.006 |
|  | BMI | -5.49e+06 | 8.78e+06 | [-2.27e+07, 1.17e+07] | -0.63 | 0.531 |
|  | Biological Sex (Female) | 6.37e+07 | 1.66e+08 | [-2.61e+08, 3.89e+08] | 0.38 | 0.701 |
|  | Age | 3.76e+06 | 2.82e+06 | [-1.76e+06, 9.29e+06] | 1.33 | 0.182 |
|  | Centiloid | -9.26e+05 | 8.16e+05 | [-2.52e+06, 6.73e+05] | -1.14 | 0.256 |
|  | Trisomy Type (Full Trisomy) | 5.10e+07 | 3.42e+07 | [-1.60e+07, 1.18e+08] | 1.49 | 0.135 |
|  | BMI x Biological Sex | 2.68e+06 | 5.09e+06 | [-7.29e+06, 1.26e+07] | 0.53 | 0.599 |
| **B2M** | **Fixed Effects** |  |  |  |  |  |
|  | Intercept | 1.80e+06 | 2.31e+06 | [-2.73e+06, 6.34e+06] | 0.78 | 0.435 |
|  | BMI | 8293.335 | 68113.440 | [-1.25e+05, 1.42e+05] | 0.12 | 0.903 |
|  | Biological Sex (Female) | -1.50e+06 | 1.28e+06 | [-4.01e+06, 1.01e+06] | -1.17 | 0.242 |
|  | Age | 1.09e+05 | 20793.626 | [67750.838, 1.49e+05] | 5.22 | <0.001 |
|  | Centiloid | -5655.444 | 6152.505 | [-17714.353, 6403.465] | -0.92 | 0.358 |
|  | Trisomy Type (Full Trisomy) | 46272.233 | 2.73e+05 | [-4.88e+05, 5.81e+05] | 0.17 | 0.865 |
|  | BMI x Biological Sex | 58522.760 | 39355.730 | [-18614.472, 1.36e+05] | 1.49 | 0.137 |
| **IL-18** | **Fixed Effects** |  |  |  |  |  |
|  | Intercept | 100.738 | 48.433 | [5.809, 195.667] | 2.08 | 0.039 |
|  | BMI | 0.037 | 1.419 | [-2.744, 2.819] | 0.03 | 0.979 |
|  | Biological Sex (Female) | -31.806 | 26.721 | [-84.178, 20.566] | -1.19 | 0.236 |
|  | Age | -0.053 | 0.431 | [-0.897, 0.792] | -0.12 | 0.903 |
|  | Centiloid | 0.157 | 0.129 | [-0.096, 0.409] | 1.22 | 0.225 |
|  | Trisomy Type (Full Trisomy) | 2.831 | 5.549 | [-8.045, 13.707] | 0.51 | 0.611 |
|  | BMI x Biological Sex | 0.920 | 0.821 | [-0.689, 2.529] | 1.12 | 0.264 |
| **sICAM-1** | **Fixed Effects** |  |  |  |  |  |
|  | Intercept | 3.02e+05 | 1.12e+05 | [81835.625, 5.22e+05] | 2.69 | 0.008 |
|  | BMI | -767.716 | 3284.152 | [-7204.654, 5669.221] | -0.23 | 0.815 |
|  | Biological Sex (Female) | -70217.803 | 62342.285 | [-1.92e+05, 51973.076] | -1.13 | 0.262 |
|  | Age | 1661.451 | 1001.502 | [-301.492, 3624.395] | 1.66 | 0.099 |
|  | Centiloid | -440.400 | 298.638 | [-1025.730, 144.930] | -1.47 | 0.142 |
|  | Trisomy Type (Full Trisomy) | 946.271 | 12808.328 | [-24158.052, 26050.593] | 0.07 | 0.941 |
|  | BMI x Biological Sex | 2505.120 | 1916.320 | [-1250.867, 6261.107] | 1.31 | 0.193 |

Note. B=unstandardized regression coefficient; SE=standard error; CI=confidence interval. Large values are displayed in scientific notation for readability. All models corrected for multiple comparisons using the false discovery rate (Benjamini–Hochberg, 1995). Sex reference = Male; site included as a random intercept. BMI=Body Mass Index; CRP=C-reactive Protein; IL=interleukin; TNF- α=Tumor Necrosis Factor–Alpha; A2M=alpha-2 macroglobulin; B2M=beta-2 macroglobulin; sICAM-1=soluble intercellular adhesion molecule-1.
